# A scoping review of the public health impact of vitamin D-fortified dairy products for fracture prevention

**DOI:** 10.1007/s11657-017-0352-1

**Published:** 2017-06-21

**Authors:** Mickael Hiligsmann, Audrey Neuprez, Fanny Buckinx, Médéa Locquet, Jean-Yves Reginster

**Affiliations:** 1 Department of Health Services Research, CAPHRI Care and Public Health Research Institute, Maastricht University, P.O. Box 616, 6200 MD Maastricht, the Netherlands; 2 Department of Public Health, Epidemiology and Health Economics, University of Liège, Liège, Belgium

**Keywords:** Cost-effectiveness, Dairy products, Fractures, Nutrition, Osteoporosis, Public health

## Abstract

**Purpose:**

Dairy products are rich in nutrients that positively influence bone health and hence fracture risk, and have therefore been recommended and used for fracture prevention. To help decision makers to efficiently allocate scare resources, it is further important to assess the public health and economic impact of any health intervention. In recent years, several studies have been conducted to estimate the public health and/or economic impact of dairy products but no overview is currently available. This article aims therefore to summarize evidence and review articles that estimated the public health and/or economic impact of vitamin D-fortified dairy products for fracture prevention.

**Methods:**

A literature review was conducted using PubMed to identify original studies that assessed the public health and/or economic impact of dairy products (or of calcium/vitamin D supplementation) for fracture prevention up to January 15, 2017.

**Results:**

Seven articles were identified. Different strategies were used by the authors to model the economic/public health impact of dairy products. The four studies assessing the public health impact of dairy products revealed a substantial benefit in terms of fracture prevented, life years, disability-adjusted life years and/or quality-adjusted life years gained. Studies assessing the cost-effectiveness revealed that the use of dairy products is generally cost-effective in the general population aged above 70 years, and from the age of 60 years in populations at high risk of fractures.

**Conclusion:**

This systematic review suggests that the use of dairy products could substantially reduce the burden of osteoporotic fractures and seem to be an economically beneficial strategy.

## Introduction

Calcium and vitamin D are essential to protect bone and prevent osteoporotic fractures. A recent Cochrane review suggests that there is high-quality evidence that vitamin D plus calcium result in small and significant reductions in the risk of hip fracture (16%), in the risk of vertebral fracture (14%) and in the risk of any non-vertebral fracture (11%) (1). International experts groups have reviewed the benefits of calcium and vitamin D for fracture prevention and provided recommendations for an optimal intake (2–6). All these articles recommend the concomitant use of calcium and vitamin D supplementation in elderly especially in patients at high risk of calcium and vitamin D insufficiency. It is generally recommended to intake 800 IU/day of vitamin D and 1000 mg of calcium per day (3).

There is high prevalence of vitamin D and calcium insufficiency in elderly men and women (7, 8). In a study conducted in nine European countries, the prevalence of vitamin D inadequacy was estimated at 80.9 and 44.5% when considering cut-offs of 75 and 50 nmol/L in elderly women aged over 80 years (8). The adequate intake of calcium is also far from optimal, with about only 20% of women consuming ≥1000 mg of calcium per day (7, 9). It is therefore needed to adequately supplement elderly patients with calcium and vitamin D especially those at high risk of vitamin D and calcium insufficiency and those taking osteoporosis medications (4, 10). Dairy products are rich in nutrients that are essential for good bone health, including calcium, vitamin D, protein, potassium, phosphorus, and other micronutrients and macronutrients (11) that are known to positively influence bone and muscle homeostasis, hence fracture risk. The European guidance for the diagnosis and management of osteoporosis (12) therefore recommends dietary sources of calcium as the preferred option. As calcium is mainly provided in dairies, calcium- and vitamin D-fortified dairy products (yogurt, milk) providing 400 mg of calcium and 200 IU of vitamin D per portion are valuable options (12).

To help decision makers in making health policy about preventive nutrition programs, it is important to assess the public health and economic impact of the intake of vitamin D-fortified dairy products. Economic evaluations are nowadays increasingly used by decision makers when making decisions about healthcare resource allocation. Given the increasing awareness of the benefits of calcium and vitamin D to prevent bone loss and the need for economic assessment, several studies have thus been conducted to assess the public health and/or economic impact of dairy products for fracture prevention in recent years. To our knowledge, no overview of these studies is currently available. Synthesizing and reviewing this literature is important to inform decision makers about the potential public health and economic impact of dairy products. The aim of this study was therefore to summarize evidence and review articles that estimated the public health and/or economic impact of vitamin D-fortified dairy products for fracture prevention.

## Methods

We conducted a review in PubMed to identify published studies that assessed the public health and/or economic impact of dairy products for fracture prevention. We combined different key terms (using MESH terms when possible) related to osteoporosis (i.e. osteoporosis, bone fracture), with economic terms (i.e. cost-benefit, cost-effectiveness, economic, cost, public health) and with nutrition terms (i.e. dairy products, nutrition, calcium and vitamin D supplementation). All original articles published in English or French until January 15, 2017, were included. We only included original research that assessed the public health and/or the economic impact of fortified dairy products for bone prevention loss. Studies assessing calcium and vitamin D supplementation were also included, while studies looking at vitamin D supplementation only were excluded. Abstract and title screening was initially performed by one investigator, followed by a full-text screening. References of identified articles were searched for additional articles; the option “see all related” from PubMed was also used and further completed by authors’ knowledge of the published literature.

Data were collected on study first author, title, country, publication year, method, outcome, interventions, and main results. Given the heterogeneity between studies, a narrative review was used to report the findings.

### Public health impact

To measure the public health impact of dairy products, different outcomes could be used including the number of fractures prevented, the number of life years saved, the number of quality-adjusted life years (QALY) or disability-adjusted life years (DALY) gained or in money (referred as cost-of-illness study). QALY is an attractive outcome measure combining quality of life (morbidity) and quantity of life (mortality) in a single metric and is also the outcome used in cost-utility analysis (13). DALY is another measure of disease burden, expressed as the number of years lost due to disability or early death.

### Economic impact

To assess the economic impact of an intervention, economic evaluations are conducted with the aim to compare the costs and outcomes of two or more health interventions (13). We distinguish between a cost-effectiveness analysis (CEA) where the outcome is expressed in natural units (such as the number of fractures prevented) and a cost-utility analysis (CUA) where QALY is used as outcome. The latter is often preferred since it allows comparisons between different interventions and diseases. The results of an economic evaluation (CUA or CEA) are expressed in terms of incremental cost-effectiveness ratio (ICER) which is defined as the difference in cost between the intervention and the comparator divided by their differences in outcomes. An ICER represents the additional cost of the intervention per unit of effect (e.g. cost per fractures prevented, or cost per QALY gained). If the ICER is lower than a certain threshold representing the maximum decision makers that are willing to pay for a unit of effect, the intervention is considered cost-effective. In CEA, no thresholds are generally available; for CUA, there is no consensus on the cost per QALY gained that represents good value for money. Thresholds in the range of €30,000–€45,000 are the most commonly used for defining cost-effectiveness. The World Health Organization has suggested a value of three times the gross domestic product (GDP) per capita as the DALY value to be used as cost-effectiveness threshold in developed countries. Borgström et al. (14) have suggested a threshold for QALY equal to two times the GDP per capital for industrialized countries. This threshold has been used for defining fracture risk thresholds in several countries (15, 16)**.**

## Results

### Study characteristics

**Fig. 1:**
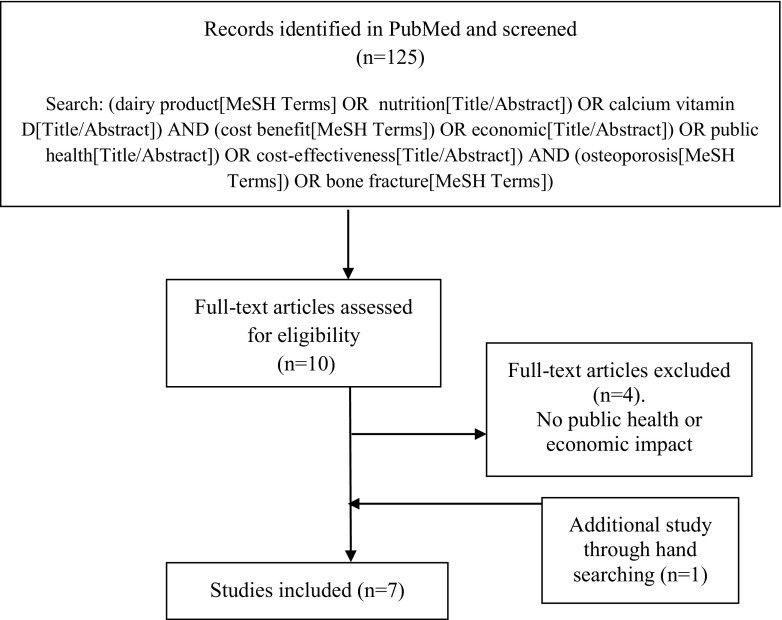
Literature search flow chart

A total of 125 articles were identified through the PubMed search, of which a total of 7 articles met our inclusion criteria (17–23) (see flow chart on Fig. 1). The study characteristics are included in Table 1.

**Table 1:** Characteristics of studies assessing the public health and/or economic impact of dairy products for fracture prevention

First author	Country	Publication year	Method	Outcomes	Intervention	Intervention effects
1. Lotters	Netherlands, France, Sweden	2013	Model	PH: costs, number of hip fractures, DALY	Increasing dairy foods consumption	Not reported
2. Hiligsmann	Belgium	2015	Markov microsimulation model	CE: cost per QALY gained	Calcium and vitamin D supplementation (compared with no treatment)	18% hip; 13% VFX; 20% other
3. Ethgen	Belgium	2015	Population-based model (using Markov microsimulation model)	PH: fractures avoided, life years gained; CE: cost per fracture avoided; cost per life year	Daily administration of one, two, or three portions of a yogurt fortified with vitamin D	18% hip; 13% VFX; 20% other
4. Ethgen	Belgium	2016	Markov microsimulation model	CE: cost per QALY gained	Daily administration of one, two, or three portions of a yogurt fortified with vitamin D	18% hip; 13% VFX; 20% other
5. Sandmann	Germany	2016	Spreadsheet-based model	PH: costs, fracture prevented; CE: benefit-cost ratio	Food-fortification programmes	9% or 25% hip; 14% VFX; 11% other
6. Hagen	Norway	2016	Markov model	CE: cost per QALY gained	Calcium and vitamin D supplementation (compared with no treatment)	16% hip; 11% VFX; 15% other
7. Hiligsmann	France	2017	Markov microsimulation model	PH: fractures prevented, QALY gained, life years gained; CE: cost per QALY gained	Recommended intake of vitamin D-fortified dairy products (2 products per day in base case)	16% hip; 14% VFX; 11% other

*CE* cost-effectiveness, *DALY* disability-adjusted life years, *PH* public health, *QALY* quality-adjusted life years, *VFX* vertebral fracture

Five of these studies (17, 18, 21–23) assessed the public health and/economic impact of dairy products (also called vitamin D-fortified dairy products, vitamin D and calcium food fortification, vitamin D-rich dairy products) while two studies (19, 20) assessed the impact of calcium and vitamin D supplementation without mentioning the specific use of dairy products. Four studies assessed the public health impact of dairy products in terms of costs, number of fractures prevented, life years gained, DALY gained or QALY gained (17, 21–23) and all studies except the study of Lotters assessed the cost-effectiveness of dairy products (or calcium and vitamin D supplementation). Four studies estimated the cost per QALY gained of dairy products (17, 19–21), one assessed the cost per fracture prevented and cost per life year gained (17), and one study used a benefit-cost ratio comparing costs and fractures prevented (23).

Three studies were conducted in Belgium (17, 18, 20), one in France (21), one in Germany (23), one in Norway (19) and one study included three countries (i.e. Sweden, the Netherlands and France) (22). All studies used a model to simulate the public health or economic impact. Most studies (17, 18, 20, 21) used a previously validated Markov microsimulation model that has frequently been used to assess the cost-effectiveness of osteoporosis interventions (24).

Different strategies were used by the authors to model the public health impact of dairy products. Ethgen et al. (17) assessed the public health impact of appropriate daily intake during the remaining lifetime of a cohort, while Hiligsmann et al. (21) assumed an appropriate intake for 1 year and assessed the lifetime implications of this 1-year appropriate intake. In Sandmann et al. (23), the effect of a voluntary food fortification programme was assessed (assuming an 82% adherence level to the programme). Lotters et al. (22) assessed the potential impact of increasing dairy foods consumption in people with an inadequate calcium intake. Hagen et al. (19) assessed the impact of cardiovascular effects on the cost-effectiveness of calcium and vitamin D supplementation.

The effect of dairy products on the risk of fractures was derived from systematic reviews. Three studies (17, 18, 20) used the results of a literature search of articles describing the efficacy of vitamin D in combination with calcium in terms of fracture risk reduction (20). The three most recent studies (19, 21, 23) used the effect of calcium and vitamin D supplementation on fracture risk from a Cochrane review (1).

### Public health impact

Four studies assessed the public health impact of dairy products (18, 21–23) (see Table 2).

**Table 2:** Public health impact of the use of dairy products for fracture prevention

Study	Public health impact
Lotters (2013)	- Yearly number of hip fractures prevented: 2023 (FR), 455 (SW), 132 (NL) - Yearly number of DALYs gained: 6263 (FR), 1246 (SW), 374 (NL) - Yearly total costs avoided: €129 million (FR), €34 (SW), €6 (NL).
Ethgen (2015)	Lifetime impact of recommended intake of dairy products over the remaining lifetime: - Number of fractures prevented: 30,376 (in women) and 16,105 (in men) - Life years gained: 6605 (in women) and 6144 (in men).
Sandmann (2016)	Vitamin D and calcium food-fortification programme in the German female population aged 65 years and older would lead to: - Annual net cost savings of €315 million - Annual total number of fractures prevented: 36,705
Hiligsmann (2017)	Lifetime impact of recommended intake of dairy products in the general French population for 1 year (2015): - Total number of all fractures prevented: 64,392 (including 19,500 hip) - 32,569 QALY gained and 29,169 life years gained.

*DALY* disability-adjusted life years, *FR* France, *NL* Netherlands, *QALY* quality-adjusted life years, *SW* Switzerland

First, Lotters et al. (22) estimated that the number of hip fractures that may potentially be prevented each year with additional dairy products was 2023 in France, 455 in Sweden and 132 in the Netherlands. The yearly number of DALY lost was estimated at 6263 for France, 1246 for Sweden, and 374 for The Netherlands. The corresponding total costs that might potentially be avoided were estimated at €129 million, €34 million, and €6 million, in these countries, respectively.

Second, Ethgen et al. (17) estimated the projected health and economic impact of the recommended dairy daily intake versus the absence of appropriate intake in Belgium. The authors estimated that 30,376 fractures and 6605 life years could be saved for an appropriate dairy intake over the remaining lifetime of all women aged over 50 years. In men, the number of fracture avoided and life years gained were estimated at 16,105 and 6144 respectively.

Third, Sandmann et al. (23) estimated that the implementation of a vitamin D and calcium food fortification programme would lead to annual net cost savings of €315 million and the prevention of 36,705 fractures in the German female population aged 65 years and older.

Fourth, in the general French population aged over 60 years, Hiligsmann et al. estimated the lifetime health impacts of the recommended intake of dairy products in the general French population for 1 year (2015) (21). The recommended intake of dairy would reduce the total lifetime number of fractures by 64,932, of which 46,472 and 18,460 would occur in women and men, respectively. In particular, 19,500 hip fractures could be prevented. Dairy products would also result in 32,569 QALYs and 29,169 life years gained.

In summary, all these studies reported a substantial public health benefit of dairy products in terms of fractures prevented as well as in life years, QALY or DALY gains. Direct comparison of studies is difficult given the variety in methodology and outcomes used. We could however mention that the benefits in terms of fractures ranged from 132 hip fractures per year in the Netherlands with additional dairy products to a lifetime gained of 63,392 fractures in France for 1 year of the recommended intake of dairy products.

### Economic impact

Six studies assessed the cost-effectiveness of dairy products (17–21, 23) (see Table 3).

**Table 3:** Economic impact of the use of dairy products for fracture prevention

Study	Cost-effectiveness
Ethgen (2015)	Using cost per fracture avoided as outcome, dairy products at a yearly cost of €350 are cost-effective from 70 years on in the general population and from 60 years on in patients at increased risk of osteoporotic fractures
Hiligsmann (2015)	The cost per QALY gained of vitamin D/calcium supplementation was estimated at €40,578 and €23,477 in women and men aged 60 years, respectively. These values were €7912 and €10,250 at the age of 70 years and vitamin D and calcium supplementation was cost-saving at the age of 80 years
Ethgen (2016)	The daily intake of two yogurts is cost-effective above 80 years in the general population and above 70 years in women at increased risk of fractures
Sandmann (2016)	Vitamin D and calcium food fortification programme is cost-saving (annual net cost savings and better outcomes resulting from fracture prevention)
Hagen (2016)	The cost-effectiveness of calcium and vitamin D supplementation was estimated at €14,453 per QALY gained for the average 65-year-old Norwegian women assuming no cardiovascular effects
Hiligsmann (2017)	The cost per QALY gained of appropriate dairy intake (2 dairy products per day) was estimated at €58,244 in the general French population aged over 60 years and fall below a threshold of €30,000 per QALY gained in women over 70 years and in men over 80 years

*QALY* quality-adjusted life years

The studies assessing the cost-effectiveness of calcium and vitamin D supplementation without referring to dairy products suggested that that the intervention is cost-effective in populations above 60 years with cost per QALY gained lower than €45,000 (19, 20). The study of Hagen et al. (19) revealed however that the potential cardiovascular effects of calcium and vitamin D supplementation could alter this conclusion.

The studies assessing directly the cost per QALY gained of dairy products suggested that the intervention is generally cost-effective in the general population aged above 70 years (21) or 80 years (17, 18) and from the age of 60 years in population at increased risk of fractures. The study of Sandmann et al. even suggested that the implementation of a vitamin D and calcium food fortification programme is cost-saving (i.e. intervention cost lower than saved costs resulting from less fractures) in the German female population above the age of 65 years.

## Discussion

This systematic review identified seven studies that assessed the public health and/or economic impact of vitamin D-fortified dairy products (or of calcium and vitamin D supplementation). Different strategies were used by the authors to model the public health impact of dairy products making direct comparison between studies difficult. The four studies assessing the public health impact of dairy products revealed a substantial potential gain of dairy products in terms of fracture prevented, life years and DALY or QALY gained. By example, in France, the appropriate intake of dairy products in the year 2015 would lead to the lifetime prevention of 64,392 fractures including 19,500 at the hip. Given 65,697 hip fractures occurred in the year 2013 in France (25), the potential benefits of dairy products are therefore substantial. Studies assessing the cost per QALY gained of dairy products revealed that the use of dairy products is generally be cost-effective in the general population aged above 70 years and from the age of 60 years in populations at high risk of fractures.

Our review therefore suggests that an adequate intake of calcium and vitamin D supplementation by the administration of dairy products could lead to a substantial decrease in the burden of osteoporotic fractures (in terms of fractures prevented, life years and QALY gained) and is cost-effective in the general population above 70 years. This review could provide relevant information for policy makers and clinicians and help them to efficiently allocate resources. Our study recommends the implementation of programmes to increase the intake of dairy products by policy makers. Clinicians should also take actions and discuss with their patients the benefits of calcium and vitamin D, to improve the consumption of dairy products.

There are some potential limitations to our review. First, we did not perform a complete systematic review following the PRISMA statement. Only one reviewer searched the literature and extracted data, and the search was also limited to one database. However, backward and forward referencing was conducted and the option “see all related” from PubMed was used for the identified article. Second, given the diversity in study methodology and outcomes, we were not able to perform a direct comparison between studies. Third, we did not provide a quality assessment of the economic studies.

We also identified some limitations of current studies and areas for further research. First, all studies assumed the efficacy of calcium and vitamin D derived from the literature, but not specific to dairy products. There are currently no studies directly estimating the effects of vitamin D-fortified dairy products on fracture prevention. Future research should therefore collect longitudinal population data for documenting the effects and net benefits of increasing dairy consumption on bone health and on the related utilization of healthcare resources (19). Second, studies would also be needed to collect additional benefits/side effects of dairy products that could potentially affect the economic benefits (19). Such effects have been poorly investigated and thus often not included in previous modelling studies. Third, further work needs to be done on adherence to dairy products. Relatively few studies have assessed the willingness of people to consume dairy products. Although the study of Sandmann et al. (26) suggests that a large majority of Germans would be willing to consume vitamin D-fortified dairy products, further work needs to be done about acceptance and adherence to fortified dairy products, and the potential effects on bone prevention. Finally, we observed a large diversity in the methodology and outcomes used to assess the effects of dairy products making comparison between studies difficult. Further guidance/recommendations needs to be done on how to model the public health and economic impact of nutritional products.

In conclusion, this systematic review suggests that the use of vitamin D-fortified dairy products could substantially reduce the burden of osteoporotic fractures and seem to be an economically beneficial strategy.
